# Biomimetic Nanostructure Platform for Cancer Diagnosis Based on Tumor Biomarkers

**DOI:** 10.3389/fbioe.2021.687664

**Published:** 2021-07-15

**Authors:** Xiping He, Yifan Ma, Haotian Xie, Gaofeng Rao, Zhaogang Yang, Jingjing Zhang, Zhong Feng

**Affiliations:** ^1^Department of Rehabilitation Medicine, The Affiliated Wenling Hospital of Wenzhou Medical University, Wenling, China; ^2^Department of Chemical and Biomolecular Engineering, The Ohio State University, Columbus, OH, United States; ^3^Department of Mathematics, The Ohio State University, Columbus, OH, United States; ^4^Department of Radiation Oncology, University of Texas Southwestern Medical Center, Dallas, TX, United States; ^5^Department of Neurology, The Affiliated Wenling Hospital of Wenzhou Medical University, Wenling, China

**Keywords:** biomimetic, nanotechnology, cancer, diagnosis, tumor biomarkers

## Abstract

Biomarker discovery and its clinical use have attracted considerable attention since early cancer diagnosis can significantly decrease mortality. Cancer biomarkers include a wide range of biomolecules, such as nucleic acids, proteins, metabolites, sugars, and cytogenetic substances present in human biofluids. Except for free-circulating biomarkers, tumor-extracellular vesicles (tEVs) and circulating tumor cells (CTCs) can serve as biomarkers for the diagnosis and prognosis of various cancers. Considering the potential of tumor biomarkers in clinical settings, several bioinspired detection systems based on nanotechnologies are in the spotlight for detection. However, tremendous challenges remain in detection because of massive contamination, unstable signal-to-noise ratios due to heterogeneity, nonspecific bindings, or a lack of efficient amplification. To date, many approaches are under development to improve the sensitivity and specificity of tumor biomarker isolation and detection. Particularly, the exploration of natural materials in biological frames has encouraged researchers to develop new bioinspired and biomimetic nanostructures, which can mimic the natural processes to facilitate biomarker capture and detection in clinical settings. These platforms have substantial influence in biomedical applications, owing to their capture ability, significant contrast increase, high sensitivity, and specificity. In this review, we first describe the potential of tumor biomarkers in a liquid biopsy and then provide an overview of the progress of biomimetic nanostructure platforms to isolate and detect tumor biomarkers, including *in vitro* and *in vivo* studies. Capture efficiency, scale, amplification, sensitivity, and specificity are the criteria that will be further discussed for evaluating the capability of platforms. Bioinspired and biomimetic systems appear to have a bright future to settle obstacles encountered in tumor biomarker detection, thus enhancing effective cancer diagnosis.

## Introduction

Compared with common medical imaging and biopsies, liquid biopsies that are noninvasive, sensitive, and capable of repeat sampling have generated considerable attention in cancer diagnosis, prognosis, and therapy (Vaidyanathan et al., 2019). Circulating tumor biomarkers, including DNA, RNA, proteins, enzymes, metabolites, and receptors, carry clinical information that can be used for liquid biopsies (Brock et al., 2015; Yáñez et al., 2015). Besides free circulating tumor substances, extracellular vesicles (EVs) (Ma et al., 2019; Walters et al., 2019, 2021) and circulating tumor cells (CTCs) contain more specific clinical information obtained from their tumor cell/tissue origin, have longer circulating time, and are desirable for diagnosis (Zhang et al., 2017). Various advances have recently been achieved for reliable, effective, efficient, and cost-effective detection platforms that specifically target protein antigens or nucleic acids, including Western blotting (WB), PCR, next-generation sequencing, mass spectroscopy, surface-enhanced Raman spectroscopy (SERS), electrochemical assay, a colorimetric assay, flow cytometry, and ELISA (Veziroglu and Mias, 2020). However, traditional ELISA may suffer from limited analytical capability due to low sensitivity, require large sample volumes, and long processing time, thus automated chemiluminescent ELISA that is capable of rapid and sensitive detection with minimum sample volume has obtained great attention (Tan et al., 2018, 2019). For example, Tan et al. detected a panel of bladder cancer-associated biomarkers (i.e., EGFR, HER2, ADAM1, Survivin), with only 8 μL of sample per marker in a rapid manner with a detection limit as low as 2 pg/ml (Tan et al., 2020). Recently, the same group has detected cancer-related EV membrane proteins successfully in an hour with high sensitivity and a minimal amount of input protein (<40 ng/marker) (Tan et al., 2021). Besides, research has shown the automated ELISA can detect clinically relevant proteins [e.g., prostate-specific antigen (PSA)] in serum from patients (Rissin et al., 2010) with prostate cancer. Moreover, surface Plasmon resonance (SPR) and localized SPR (LSPR) are widely used for cancer biomarkers in a liquid biopsy. Monteiro et al. have detected the human epidermal receptor protein-2 (HER2), using SPR (Monteiro et al., 2016). Immunostaining (including a conjugated fluorescence antibody, aptamer, a molecular beacon) platforms (e.g., microarrays, microfluidics, and chips) can also reveal clinical information (Lau et al., 2010; Davies et al., 2012; Yoo et al., 2012; Ko et al., 2020; Rima et al., 2020). Though these platforms exhibit robustness, most of them suffer from unsatisfactory accuracy and low sensitivity and specificity due to the heterogeneity of substances, a dearth of biomarkers, and contamination (Willms et al., 2018). Current methods are also limited by the laboratory scale as opposed to authentic real clinical applications. Hence, platforms that are capable of efficient capture, a low dose, signal amplification, background elimination, cost-effective, rapid, high-throughput, user-friendly, and multiple and multiplexed analysis are essential.

Biomimetic and bioinspired technology has gained considerable attention in biomedical practice due to its unique properties (Naik and Singamaneni, 2017; Chen et al., 2019; Liu H. et al., 2019). For example, photonic crystals (PCs) inspired by natural photonic nanostructures can control light and are suitable for signal amplification (Lopez, 2003). Combining PCs with ELISA, single-strand DNA, quantum dots, and other materials that can recognize tumor biomarkers enhances the detected signal and reduces background noise, thus improving sensitivity. Other materials, such as silicon nitride, alumina membranes, and polymer membranes used for fabricating nano channels to mimetic cell membranes, can better regulate targets and transfer signals, leading to sensitive diagnostics (Holt et al., 2004; La Flamme et al., 2007; Kochkodan and Hilal, 2015). Beyond nano channel fabrication, coating nanoparticles with cell membranes or vesicles are also applicable (Zhu et al., 2016). Additionally, inserting living organisms into the synthesis process to produce sophisticated structures with unique natural physical and chemical properties, such as nanoparticles or nanoflowers, can enhance the capture efficiency for tumor targets because of their large surface-to-volume ratio and hollow structures (Heller and Forkmann, 1988; Mohanpuria et al., 2008). Beyond capture efficiency, functional groups, such as illuminance materials, can be encapsulated in hollow structures and enhance detection signals (Dong et al., 2020). Moreover, special structures inspired by animals/plants can also be used for biosensors (Wu et al., 2017; Iravani and Varma, 2019). For instance, the cactus spine is a good candidate for controlling droplet transportation in open microfluidics (Wu et al., 2019). Also, influenced by an octopus, pathogen detection with high efficiency was accomplished (Lv et al., 2019).

Thus, in this review, we focus on the recent progress of biomimetic platforms for cancer diagnosis. We first introduce and summarize tumor biomarkers and their important roles in cancer diagnostic settings. Next, different biomimetic systems, including PCs, nanochannels, bioinspired nanoparticles, nanoflowers, cell membrane-based systems, animal/plant-inspired systems, and their principles and achievements, are introduced. We also compare and summarize the disadvantages and advantages of these platforms. Challenges and future perspectives are also covered.

## Tumor Biomarkers

### Clinical Relevance of Free Antigens/Proteins in Cancer

Various studies have been conducted to demonstrate the clinical relevance of specific proteins in different cancers. For example, carbohydrate antigen and 125 (CA-125), a U.S. Food and Drug Administration (FDA)-approved cancer biomarker, has been used clinically for monitoring ovarian cancer therapy, diagnosis, prognosis, and detecting recurrence (Moss et al., 2005). Soltész et al. collected both tissue and blood samples from ovarian cancer cases and showed that CD24 was overexpressed in cell-free plasma, EVs, and tissue samples in epithelial ovarian cancer (Pavlou et al., 2013). As for lung cancer, the epidermal growth factor receptor (EGFR) was remarkably higher in serum samples obtained from patients (Maheswaran et al., 2008) with lung cancer. Li et al. also demonstrated the potential of EGFR in lung cancer diagnosis (Li F. et al., 2017). Moreover, EGFR and carcinoembryonic antigen (CEA) are FDA-cleared colorectal cancer biomarkers for therapy monitoring, diagnosis, prognosis, and hepatic metastases screening (Spano et al., 2005). Taking gastrointestinal and pancreatic cancer together, the conventional serum carbohydrate antigen 19-9 (CA19-9) is a potential tumor marker, and CA19-9 has been used for therapy monitoring incurrent clinical settings (Mann et al., 2000). For gastrointestinal cancer prediction, the v-kit Hardy-Zuckerman 4 feline sarcoma viral oncogene homolog (KIT) is an FDA-cleared protein biomarker (Rönnstrand and Lennartsson, 2016). Studies related to pancreatic cancer biomarkers are more complementary; glypican-1 (GPC1), MIF, EGFR are all used to successfully diagnose patients with healthy controls (Li et al., 2018). Recently, a novel biomarker named ZIP4 has been shown to promote pancreatic cancer growth (Li et al., 2007). Studies related to prostate cancer protein biomarkers are limited, but PSA is a well-known FDA-cleared diagnosis and a monitoring biomarker (Balk et al., 2003). Concerning bladder cancer, FDA-approved biomarkers, including nuclear matrix protein 22 (NPM-22), fibrinogen-degradation products, bladder tumor antigens, higher molecular, and mucin have been found upregulated in urine (Kim and Bae, 2008). HER2 is highly expressed in all stages of breast cancers and is clinically approved for trastuzumab therapy monitoring (Abubakar et al., 2019). Carbohydrate antigen 27.29 and carbohydrate antigen 15.3 can serve as therapy monitoring (Jensen et al., 1991; Bast et al., 2001). As for the glioblastoma, EGFR and EGFRvIII mutation is widely used for diagnosis, especially EGFRvIII, which correlates with all glioma subtypes (Heimberger et al., 2005).

### Clinical Relevance of Free Nucleic Acids in Cancer

Free circulating nucleic acids found in the bloodstream and other biofluids have also been examined as cancer biomarkers. The miR-21, miR-17-5p, miR-1246, miR4644, miR-3976, and miR-4306 have been upregulated in patient-healthy case-control studies with around 80% sensitivity and specificity (Que et al., 2013). Another study recruited a large cohort of patients with pancreatic cancer (*n* = 216) and showed that miR-122-5p, miR-125b-5p, miR-193b-3p were upregulated (Madhavan et al., 2015). As for prostate cancer, miR-21, miR-141, and miR-375 are highly expressed with 0.817, 0.712, and 0.707 AUC, respectively (Foj et al., 2017). Long non-coding RNA (lncRNA), such as lncRNA-p21 with AUC.663, is also significantly higher in patients (Işin et al., 2015; Foj et al., 2017; Wang et al., 2018). Combining CRNDE-h and CEA, reaching an AUC of 0.913 thus can be good biomarkers for the diagnosis and prognosis of colorectal cancer (Dragomir et al., 2018). miR-21 and miR-1246 can also be used for diagnostic and prognostic purposes for colorectal cancer (Desmond et al., 2020). Moreover, miR-23b-3p, miR-21-5p, miR-96, miR-155, miR-122, miR-146, and miR-30b/c are widely used for lung cancer (Bi et al., 2020). As for breast cancer, miR-105 and miR181-a are metastatic stage biomarkers (Li H.-Y. et al., 2017), and miR-1246 and miR-21 are general biomarkers in all stages of breast cancer (Eichelser et al., 2014). Regarding ovarian cancer, miR-373, miR-200a/b/c, miR-21, and miR214 can serve as upregulation biomarkers (Wang et al., 2019). In glioma, TIMP-1, IL-8, and ZAP70 are widely used mRNA diagnostic biomarkers (Muller et al., 2015). Furthermore, miR-301a, miR-22, miR-222, and miR-182-5p are highly expressed in glioma (Toraih et al., 2019).

### Clinical Relevance of Tumor Extracellular Vesicles (tEVs) in Cancer

Extracellular vesicles ranging from 40 nm to a few microns are nanovesicles that carry molecular contents, depending on their cell (tissue) origins (Liu Y. et al., 2019; Shi et al., 2020; Yang et al., 2020a,b, 2021; Ma et al., 2021). There are two distinct biogenesis processes of EVs: one starts with early endosome formation from the cell membrane, which produces internal vesicles through budding inward to the membrane (Kalluri, 2016) that are released after late endosome fusing with cell membranes (Sokolova et al., 2011). Another type of EV is released from the cell membrane directly (Akers et al., 2013; Shi et al., 2018). All kinds of cells release EVs regardless of whether under healthy or pathological conditions, while the tumor cells may release more abundant EVs into the extracellular microenvironment than normal cells (Hessvik and Llorente, 2018). EVs contain common characteristic proteins, such as TSG101, Alix, Tetraspanin, and Rab family proteins that belong to the endosome biogenesis process (Hessvik and Llorente, 2018). Tetraspanins (e.g., CD63, CD9, and CD81) are often used as general markers for vesicle confirmation (Bobrie et al., 2012).

Tumors can serve as plants that generate tEVs, which function like seeds in various biofluids, including blood, urine, milk, saliva, and cerebrospinal fluids (Minciacchi et al., 2015). EVs carry multiple genetic materials derived from tumors that are capable of affecting neighbor and distant organs and cells (Zhang et al., 2015). Therefore, EVs may facilitate cancer diagnosis and prognosis and monitor treatment outcomes (Alix-Panabières and Pantel, 2017). However, EVs present in human biofluids are composed of massive non-tumor biomolecules that impede downstream analysis (Andreu et al., 2016), requiring special enrichment and purification steps (Zhou et al., 2006). Current methods used to isolate EVs include differential centrifugation, density gradient centrifugation, size-exclusion chromatography, polymer precipitation, ultrafiltration, tangential flow filtration, microfluidics, and immunoaffinity (Yuana et al., 2014; Yamashita et al., 2016; Koh et al., 2018; Patel et al., 2019). After isolation, exosomal proteins or nucleic acids are characterized to provide clinical information. Sandfeld-Paulsen et al. isolated exosomes from 431 patients with lung cancer and demonstrated that CD151, CD171, and tetraspanin 8 were the strongest separators of patients with cancer with above 0.6 area under the curve (AUC) (Sandfeld-Paulsen et al., 2016). Recently, Zhang et al. have found that CD24 was upregulated compared with healthy controls in patients (Zhang et al., 2021) with metastatic breast cancer.

### Clinical Relevance of CTCs in Cancer

Circulating tumor cells are cancer cells present in the bloodstream that depart from solid tumors. CTCs are rare but contain massive tumor derivatives that are of paramount importance for cancer diagnosis. Isolation of CTCs is challenging primarily because of their rarity and viability after the isolating process. Common enrichment methods include positive selection, which captures CTCs based on their surface antigens, and negative selection, which removes all other cells [e.g., red blood cells (RBCs)] in the blood. Reátegui et al. (2015) developed the microfluidic herringbone CTC-chip fabricated with a layer-by-layer gelatin nanocoating and immunoaffinity structure to specifically capture CTCs in the bloodstream of patients with breast and lung cancer and release the captured CTCs with high efficiency using the temperature-sensitive gelatin layer (Reátegui et al., 2015). Furthermore, an FDA-approved technique, the Cell Search system, which consists of a two-step procedure, first captures the CTCs with magnetic ferrofluids functionalized with anti-epithelial cell adhesion molecule (EpCAM), and then applies anti-cytokeratin antibodies for further identification (Riethdorf et al., 2007). Positive selection provides high capture efficiency for tumor-antigen highly expressed CTCs; however, it heavily relies on the expression levels of given antigens, which vary under different conditions. Therefore, negative selection, such as size-based filtration and negative immunomagnetic depletion of white blood cells (WBCs), has been developed. Size filtration applies to the size differences between CTCs and blood cells. CTCs normally have larger diameters. Immunomagnetic selection relies on CD45, an abundant biomarker for WBCs.

## Applications of Biomimetic and Bioinspired nanoplatforms on Cancer Diagnosis

All tumor biomarkers, no matter which subtypes, require accurate platforms for characterization. Biomimetic nanoplatforms that are combined with various nanotechnologies have obtained considerable attention due to their excellent performance. In the following sections, we focus on the application of these bioinspired nanoplatforms.

### Photonic Crystals

Inspired by the photonic nanostructures of natural color materials that are able to control light, various artificial photonic materials have been designed to confine and manipulate light, allowing adjusting functions to enhance or eliminate the intensities (Parker and Townley, 2007). PCs, which contain periodic nanostructures, are one of the most widely used artificial materials in biosensors due to their excellent performance in signal amplification. In principle, the emission light is reflected in the direction of the bandgap instead of passing through the PCs surface when the emission light of fluorophores overlaps with the photonic bandgap. Thus, both the emission light of fluorophores on PCs surface and the reflected light are detected for signal amplification (Inoue and Ohtaka, 2004). Hence, PCs are good candidates to improve the sensitivity of tumor biomarker detection.

**Figure 1:**
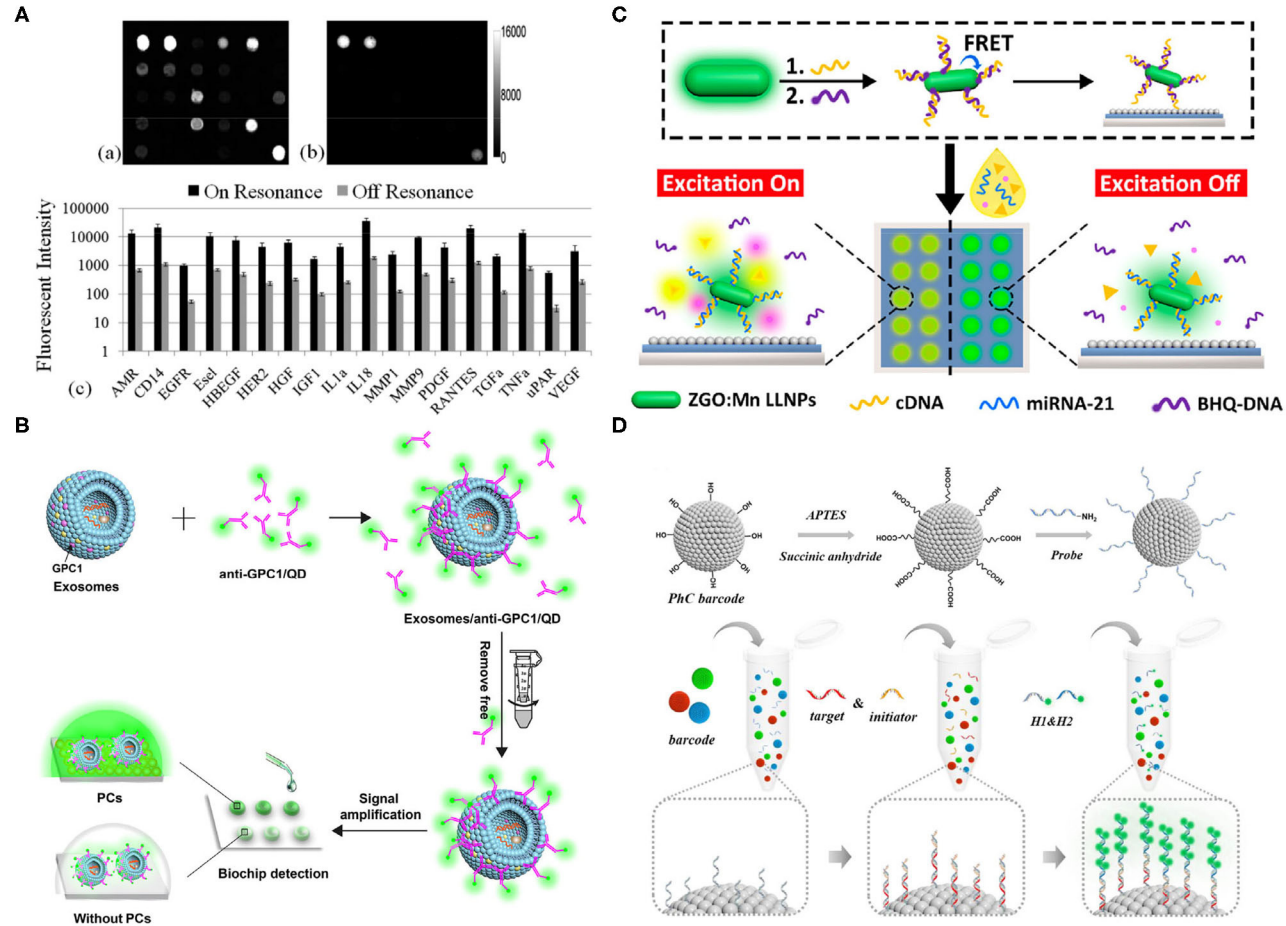
**(A)** On (left)/off (right) resonance fluorescence images when a photonic crystal (PC) array was exposed to the second-highest analyst concentration; images were taken by identical photomultiplier tube gain settings. Below is the quantitative fluorescent intensity for different functional assays, using on/off resonance; 11- to 20-fold intensity difference was observed when using the on resonance (Huang et al., 2011). **(B)** A schematic of the PC signal amplification when measuring tumor exosomes. Biotinylated anti-glypican-1 (GPC-1) antibodies were first conjugated with streptavidin-coated quantum dots, which are specifically recognized GPC1^+^ exosomes. The signal amplification was conducted by the PCs (Zhang J. et al., 2020). **(C)** A schematic of miR-21 detection, using a developed biochip. The single-stranded DNAs (ssDNAs) were functionalized onto long lifetime luminescence nanoparticles (LLNPs) and hybridized with black-hole-quencher-labeled DNAs (BHQ-DNAs), which were free after the adding of miR-21 since miR-21 attracted ssDNAs. BHQ dye-generated luminescence signals were improved by PCs (Wang et al., 2019). **(D)** A schematic of a photonic crystal (PC) barcode functionalized with amino groups and probes that target different microRNAs (miRNAs). Below is the schematic of hybridization chain reaction (HCR). The initiator chain and target chain were paired and captured on the PC barcode. Then, the hairpin fluorescence sequences were added to form a copolymer for enhancing the signal (Wei X. et al., 2020). All copyright 2021 American Chemical Society.

Huang et al. detected multiple breast cancer biomarkers (up to 24 antigens) simultaneously on a single immuno-microarray, using PCs (Figure 1A) (Huang et al., 2011). Briefly, PC-enhanced surfaces were proposed to provide resonance at the excitation wavelength to excite the fluorescent dye (cyanine-5, Cy5), resulting in an elevated electric field magnitude around 100–200 nm above the PC surface. Simultaneously, a second resonance at the wavelength of fluorophore (Cy5-labeled analytes) was generated, thus providing the enhanced photo collection efficiency and fluorescent intensity that could be detected, using commercial confocal microarray scanners. In this work, nanoreplica molding was selected for PC fabrication, and the epoxysilane-based surface chemistry was applied due to its binding capacity and low auto fluorescence background. The ELISA was used to guarantee the multiple analyses, and capture antibodies were printed on PC slides and incubated overnight at room temperature. Later, a mixture of biotinylated secondary antibodies at 25 ng/ml in PBS-Tween was added onto the array after the hybridization of analytes to avoid nonspecific interactions. Finally, 1 ug/ml SA-Cy5 was used to label the secondary antibodies. On- and off-resonance illumination was examined to validate the performance of the array; the on mode improved the signal-to-noise ratio by 3.8- to 6.6-fold and enhanced the limit of detection to <2 pg/ml. Compared with traditional ELISA, the PC surface used in this study improved the signal-to-noise ratio and increased the limits of detection (LOD) in the range of 1.3 pg/ml to 1.9 ng/ml. Simultaneous and multiplexed detection (up to 24 antigens) on a single chip also showed the robustness and high throughput of the microarray in the clinical setting. Subsequently, the same group combined a quartz-based PC with a detection instrument that contains enhanced fluorescence (EF) and label-free (LF) modalities, using collimated laser illumination for detecting 21 breast cancer biomarkers. The quartz-based PC structure, which provided low auto fluorescence, was fabricated by nanoimprint lithography in the “step-and-flash” mode, which enabled fluorescence enhancement and LF quantification of the density and variability of fixed capture antibody spots, allowing pixel-by-pixel-based resonant coupling condition optimization. The detection instrument captured the entire field of view at once, using an angle-scanning approach rather than scanning the PC surface and provided better coupling efficiency because of its collimated laser illumination. The modified chip increased the LOD with the lowest concentration at (2.1–41 pg/ml) in the mixed sample because it reduced the coefficient of variation of replicates by 20–99% compared with common fluorescence microscopy. Both studies showed the potential of PC in enhancing signals and LOD when identifying biomarkers, and their simultaneous multiplexed capabilities demonstrated high efficiency in clinical applications. However, this technology requires complicated procedures during chip fabrication, leading to low reproducibility caused by experimental and physical errors.

Quantum dots (QDs) combined with PC can also serve as a signal amplification tool for exosome diagnostic biochips. Zhang et al. took advantage of QDs with PCs to detect GPC1 with ultra-sensitivity and specificity (Figure 1B) (Zhang J. et al., 2020). The PCs used in this study, which effectively reflected emission light at 525 nm, were fabricated, using solvent evaporation. The anti-GPC1/biotin was incubated with streptavidin-labeled QDs at 160:1 at room temperature in the dark, and then incubated with exosomes at a concentration of 1.0 × 10^7^ particles/mL. Unbounded anti-GPC1/QD was washed away, using ultra centrifugal units at 100 kDa molecular weight. Finally, exosomes-GPC1/QD was captured on the PC biochip for quantitative analysis. Simple, nonenzyme involved, high sensitivity, low dose of sample usage, and acceptable reproducibility are the advantages of this chip. Moreover, the differences in mimetic serum samples and healthy controls demonstrated its potential in clinical use. Compared with WB or other traditional exosome characterization methods, this biochip shows high sensitivity and requires a minimum sample since QDs and PCs amplify signals extensively. Nevertheless, there are two aspects that can be improved, one of which is adding multiple-antigen detection, and the other of which is adding the multiplexed analysis of antigens on the same exosome for revealing heterogeneity.

Except for proteins, nucleic acid signals can also be detected and enhanced by combining probes with PCs. Wang et al. detected miR-21 in urine successfully, using an optical biochip with an amplified luminescence signal and low-background interference provided by time-gated luminescence of long lifetime luminescence nanoparticles (LLNPs) and PCs (Figure 1C) (Wang et al., 2019). In brief, single-stranded DNAs (ssDNAs) were functionalized on LLNPs and partially hybridized with black-hole-quencher-labeled DNAs (BHQDNAs), on which the luminescence could be quenched by fluorescence resonance energy transfer (FRET) occurring between LLNPs and BHQDNAs. The luminescence could be recovered once the miR-21 was added since its longer sequence is more competitive to the ssDNAs, leading to the separation of BHQDNAs from the ssDNAs. In other words, the biomolecules were “off” until the target RNAs were added to trigger the luminescence. This “off–on” mode could maintain the luminescence of the long lifetime probes, whereas allowing the rapid decay of the auto fluorescence of biomolecules, thus ensuring the ultra-low background signal. Meanwhile, the chip endowed highly sensitive miR-21 detection because of its PC resonance signal amplification capability. Overall, a remarkable detection of a limit of 26.3 fM was achieved, benefiting from the above advantages, which exhibited a superior lower detection limit compared with traditional PCR. However, simultaneous multiple miRNA characterization was not included in this study, which impedes the throughput and scale of the technology in clinical settings.

Planer microarrays typically have steric hindrance problems, causing binding difficulty. To solve this problem, Wei et al. utilized photonic crystal (PC) barcodes to alter positions in the solution randomly without harmful effects on the binding efficiency, combined with hybridization chain reaction (HCR) for high-sensitivity miRNA detection (Figure 1D) (Wei X. et al., 2020). The structure of PC barcodes provided a large surface area and binding sites for probe binding, and PC barcodes with photonic bandgaps could generate characteristic reflective peaks due to their periodic porous nanostructures, allowing multiplexed detection of miRNAs. However, the detection signal that only relied on the PC was weak. Thus, the HCR was introduced for signal amplification, and its initiator chain was paired to the target chain, and the hairpin fluorescence sequences H1 and H2 formed a DNA copolymer for signal amplification by alternate initiation. Overall, they were able to detect miRNAs (i.e., miRNA-133a, miRNA-143, and miRNA-200b) in a quantitative, multiplexed, rapid, accurate, and sensitive manner, which thus can be applied as a powerful clinical analysis platform of bladder tumors, although its low reproducibility may be a potential drawback due to the complicated fabrication procedure.

### Biomimetic Nanochannel

Nanochannels are often referred to as nanopores with pore depths much larger than the diameters (Hou and Jiang, 2009). Biological cell membranes contain numerous nanochannels that conduct regulation, material exchange, signal transfer, and energy conversion. Nanochannels have significant advantages in application due to their flexibility in size and shape. However, natural nanochannels are only present in cell membranes and have restricted clinical use. Thus, fabricating artificial nanochannels as novel biosensors is becoming the focus of attention. Silicon nitride, graphene, glass, alumina membranes, and polymer membranes have been widely used for artificial nanochannel fabrication. Fabricating with these functional molecules, which mimics the gating mechanism of biological nanochannels, provides responses that can be detected when force, light, ions, and pH are applied as stimuli (Zhang S. et al., 2020). Hence, these nanochannels can be used for biosensors and drug delivery platforms with high stability and sensitivity. For example, Zhang et al. developed a biomimetic nanochannel functionalized with N-(1- naphthyl), ethylenediamine (NEDA, guest), and γ-CD (host) for amino acid transportation detection (Zhang et al., 2019).

**Figure 2:**
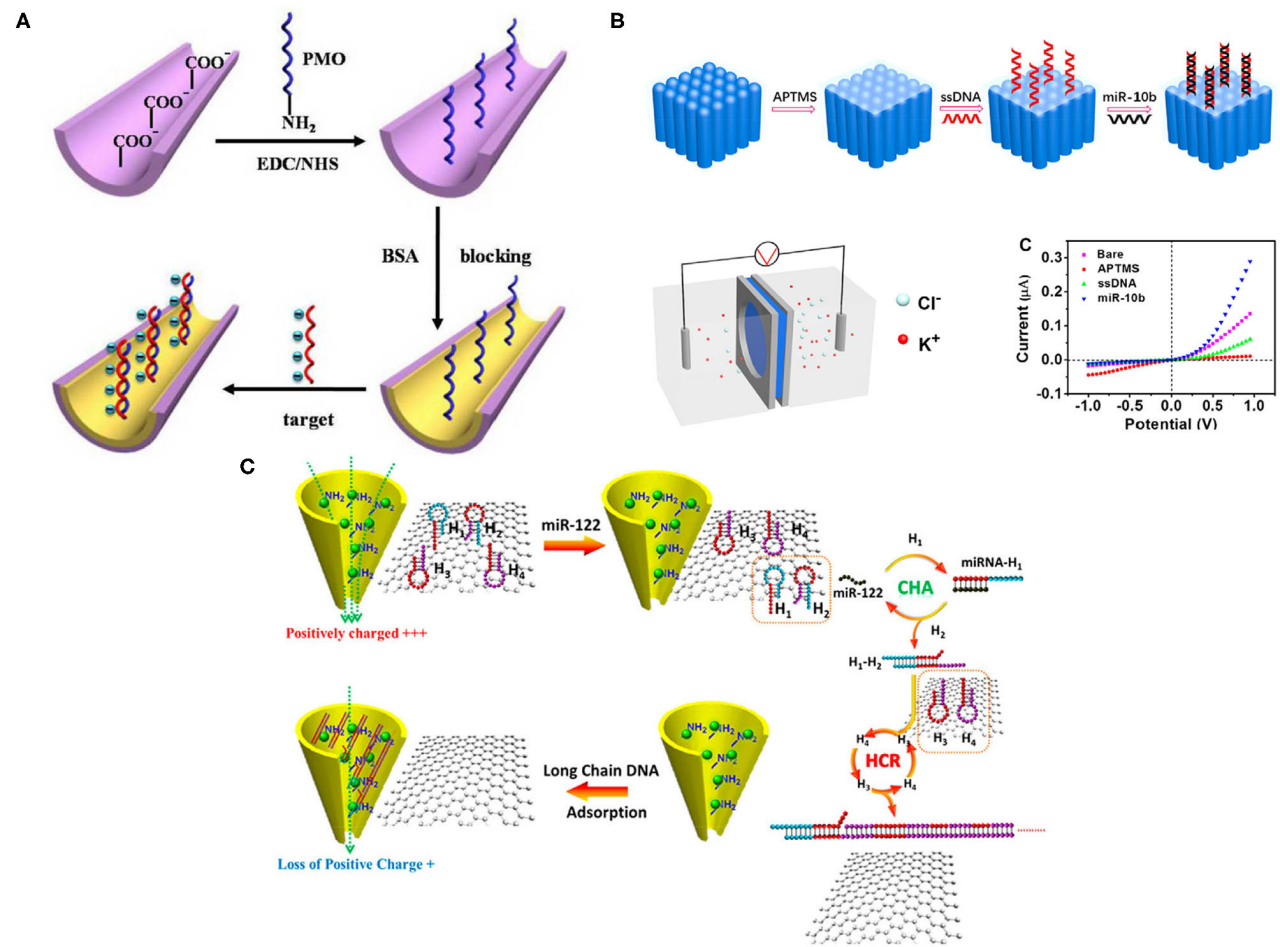
**(A)** A schematic of microRNA (miRNA) detection, using Phosphorodiamidate Morpholino Oligos (PMO)-functionalized nanochannel. Nanochannels were first prepared in Poly-ethylene terephthalate (PET); amine-modified PMO groups were then added onto nanochannel after ionized carboxylic groups modification. Bovine serum albumin (BSA) also brought more negative charges and blocked the surface. Once miRNAs were hybridized with PMO, charge density changes were detected and monitored (Liao et al., 2017). **(B)** A schematic of miRNA detection on the nanochannel-ionchannel. When miRNA was added, a single-stranded DNA (ssDNA) probe could recognize it *via* hybridization. Subsequently, the homemade electrochemical cell detected the different charge densities. The miR-10b showed the dramatic current change in the system (Zhang S. et al., 2020). **(C)** A schematic of miR-122 detection in the nanochannel. Positive charges of Zr^4+^-PEI-coated nanochannel were reduced after adding long-chain DNA due to its negative charge, allowing charge modification with a small number of targets. Once adding miR-122, it initiated multiple cycles of self-assembly between H1 and H2, generated H3 and H4, leading to lots of long ssDNAs, causing ion current changes on the nanochannel (Zhao et al., 2019). All copyright 2021 American Chemical Society.

Conical nanochannel-based biosensors have obtained substantial interest because of their ion current changes and their ion permeability, which is dependent on surface density charges. Phosphorodiamidate Morpholino Oligos (PMO), a synthetic DNA analog with a neutral backbone of morpholine rings, presents high flexibility and solubility in a solution that is suitable for surface modification in the conical nanochannel. Liao et al. anchored PMO as the capture probe on the nanochannel covalently for label-free detection of miRNAs in both PBS and serum samples (Figure 2A) (Liao et al., 2017). Once miRNAs were detected, surface charge density was observed due to the PMO/miRNAs hybridization reaction; the efficiency of which was enhanced because of the neutral character and high sequence-specific affinity of PMO. Consequently, the background noise was reduced, and the detection specificity and sensitivity were improved. Let-7b was specifically discriminated from miR-21 and Let-7c, using the PMO-modified nanochannel, and the sensitivity reached 1 fM in PBS and 10 fM in the serum sample, respectively, which were much lower than traditional PCR technology. Also, reversible variation experiments were conducted and showed that the biosensor has reliable reusability and reproducibility. However, multiple biomarker characterization was not implemented in this study.

Since the interior of the nanochannel is usually confined, this limits the adequate modification and assembly of the detection probe. Also, the modification process can be time-consuming and expensive due to the inner wall. Hence, Zhang et al. developed a new platform that fabricated the graphene oxide (GO) and Zr^4+^with dual-mode signal amplification, including target-catalyzed hairpin DNA assembly (CHA), coupled with HCR for label-free detection of miRNA (Figure 2B) (Zhang S. et al., 2020). In principle, this dual-mode amplification assembly is a double-stranded DNA structure, a ssDNA formed into a duplex conformation with targeted miRNA; the excess ssDNA was removed by GO. Later, the duplex structures were adsorbed onto the inner wall of the nanochannel because of the strong affinity between zirconium ions and phosphate groups with the assistance of an external electric field. Consequently, the changes of charge in the nanochannel were detected. This new platform is low-cost and sensitive, with a 97.2-aM limit of detection of target miR-122, and exhibits a wide detection range but without multiple characterizations.

Making the ionic current rectification happen on the outer surface instead of on the inner wall is another way to improve simplicity and sensitivity. Zhao et al. established a nanochannel-ionchannel hybrid platform, coupled with porous anodic alumina (PAA) membrane for miRNA recognition (Figure 2C) (Zhao et al., 2019). PAA was selected because of its chemical and mechanical stability and high-channel densities, allowing ionic current response amplification. In principle, when the target miRNA (miR-10b) was added, the ssDNA probe immobilized on the surface of ion channels was able to recognize it through hybridization, which generated changes in charge density on the outer surface and mass transport properties in the nanochannel-ionchannel hybrid that could be further detected by the homemade electrochemical cell. Using this hybrid platform, real-time and label-free detection of target miR-10b was achieved with a low detection limit of 15.4 aM, which is much lower than traditional RNA characterization methods, but the complex experimental procedures technology is a potential drawback.

### Nanoparticles

Nanoparticles, nanoflowers, nanoenzyme, and other nano-scale materials have received considerable attention since they have high stability, durability, selectivity, a large surface-to-volume ratio, simple synthesis, and low-cost in a variety of biosensors (Lee et al., 2016; Durrani-Kolarik et al., 2017; Sun et al., 2018a, 2021; Tran and Kim, 2018; Wu et al., 2020). Furthermore, combining bioinspired materials with nanotechnologies that alter or modify the properties of nanomaterials can decrease background noise, increase targeting efficiency, and enhance the detected signal.

**Figure 3:**
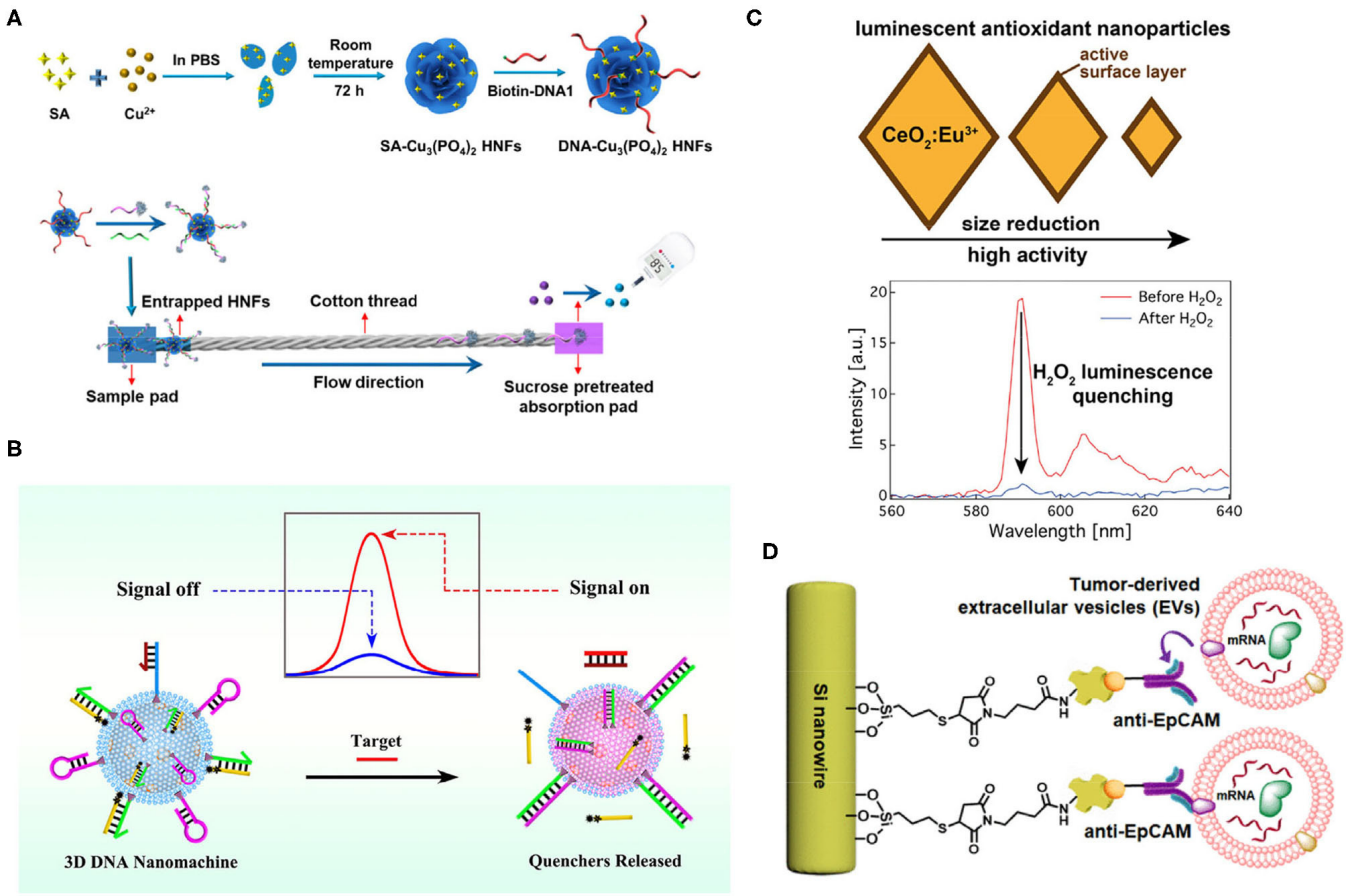
**(A)** A schematic of the formation of DNA–Cu_3_(PO_4_)_2_ hybrid nanoflowers (HNFs)s and their detection of target microRNAs (miRNAs). Target miR-21 conjugates with HNFs and trapped on the cotton thread; free DNA2-invertase conjugates hydrolyze sucrose into glucose, and the signal was readout by personal glucose meter (PGM) (Wu et al., 2018). **(B)** A schematic of the signal on and off when detecting miRNA, using 3D Nanomachine. Targets were recognized by nanomachine, which released quenchers, and the fluorescent signal was detected (Peng et al., 2019). **(C)** The smaller size of CeO_2_:Eu^3+^ demonstrated high antioxidant activity. Intensity changed at 590 nm after adding H_2_O_2_ (Pratsinis et al., 2017). **(D)** A schematic of nanowire capturing tumor extracellular vesicles (EVs). Anti-EpCAM-recognized epithelial cell adhesion molecule (EpCAM) on tumor EVs was functionalized on the Si nanowires, which captured tumor EVs efficiently due to the biomimetic structure of Si nanowires (intestinal microvilli) (Dong et al., 2019). All copyright 2021 American Chemical Society.

Wu et al. drew inspiration from living organisms to fabricate organic/inorganic hybrid materials by mixing proteins with a metal ion solution and forming DNA–Cu_3_(PO_4_)_2_ hybrid nanoflowers (HNFs) that worked as captors for miR-21 detection with a limit of.41 nM (Figure 3A) (Wu et al., 2018). Apart from generally superior properties, as with other nanomaterials, DNA–Cu_3_(PO_4_)_2_ hybrid nanoflowers (HNFs) can be readily functionalized into microfluidic channels with cotton thread for miRNA recognition. A sandwich structure “HNF-DNA1/miR-21/DNA2-invertase” was formed once miR-21 was added to the microfluidic channel. Also, unstrained NDA2-invertase was immobilized to the HNFs. During the flow transportation, large HNF complexes were adsorbed by the cotton thread while free DNA2-invertase conjugates moved to the adsorption pad and hydrolyzed sucrose fixed on the pad. Moreover, the glucose concentration was read by the personal glucose meter (PGM), which was later translated to a miR-21 concentration. By applying DNA–Cu_3_(PO_4_)_2_hybrid nanoflowers (HNFs), sensitive and specific detection of miR-21 in a rapid and cost-effective manner was accomplished. However, time-consuming procedures and the lack of multiple analyses are shortcomings.

Peng et al. combined silica@CdTe quantum dots (SiO_2_@CdTe), with lipid bilayers of small unilamellarvesicles (SUVs) and generated biomimetic SiO_2_@CdTe@SUVs, which formed a three-dimensional DNA nanomachine through the cholesterol-lipid interaction after incubation with DNA substrates for 30 min (Figure 3B) (Peng et al., 2019). This DNA nanomachine could detect miRNA-141 with a detection limit of 0.21 pM. In brief, the DNA nanomachine carried a cholesterol-labeled arm (chol-arm) with a blocker, a cholesterol-labeled hairpin (chol-H1), a cholesterol-labeled assist chain 1 (Chol-A1), and a quencher-labeled assist chain 2 (A2), and once they encounter target miRNAs, a Chol-arm was released with a blocker. Then, an opened chol-arm met with Chol-H1 and formed an arm-H1 hybrid. The opened Chol-H1 further reacted with Chol-A1 and released A2 *via* strand displacement reaction. Subsequently, after multiple rounds of strand displacement reaction, abundant A2 resulted in a strong fluorescence signal that was quantified. Hence, sensitive detection with adequate signal amplification was proposed. This platform could also detect cell lysates. Intact cells and exosomes may also be promising with permeabilization buffer.

Biomimetic artificial enzymes that possess redox potential can react with hydrogen peroxide (H_2_O_2_), leading to optical signal changes without organicdyes, and could be used for biosensing, such as ELISA. Pratsinis et al. utilized CeO_2_ nanoparticles-generated luminescence after Eu^3+^, doping for nanoenzyme synthesis, the antioxidant activity of which was highly dependent on the surface area (Figure 3C) (Pratsinis et al., 2017). H_2_O_2_ could effectively quench the luminescence of nanoenzyme, and the emission intensity at 590 nm could be observed before and after adding the H_2_O_2_. Unfortunately, this device used alcohol with a 5.2 ppb detection limit. However, combining nanoparticle antioxidant activity and rare-earth doping luminescence can also be applied for tumor biomarker detection after modification.

Inspired by the properties of intestinal microvilli, Dong et al. designed an anti-EpCAM-grafted silicon nanowire, which increased the surface area, allowing highly efficient capture of EpCAM+-EVs from plasma (Figure 3D) (Dong et al., 2019). Also, integrating with microfluidics generated a chaotic mixer and further increased the capture efficiency. Overall, an efficient and rapid platform to specifically capture tumor EVs from none-small-cell-lung cancer was proposed. Coupling with PCR, they were able to recover over 82% RNA compared with immunomagnetic beads and ultracentrifugation at 31 and 22%, respectively. Beyond these achievements, multiplexed RNA/antigen characterization to reveal heterogeneity of EpCAM-EVs can be accomplished if fixation is applied in this study.

### Cell-Membrane-Based Biomimetic Systems

Cell membrane-coated nanoparticles in biomedical applications are promising because membrane structures offer specific recognition and targeting, thus making modified nanoparticles suitable for drug delivery and diagnosis (Yang et al., 2016; Gong P. et al., 2020). For example, Khalil-Mgharbel et al. (2018) used a biomimetic lipid bilayer membrane together with spectroscopy to successfully detect a solution VE-cadherin (sVE) biomarker that is associated with kidney cancer. They proved thats VE was carried by lipoproteins, especially by high-density lipoprotein (HDL) in the blood, and that sVE could react with the lipidic membrane (adsorption) by applying electrical impedance spectroscopy (EIS) measurements of the functional changes in lipid bilayer membranes if sVE was adsorbed onto the membranes; the thickness of the lipid bilayer could be observed. The biomimetic device was capable of reproducing a lipid bilayer membrane that was tethered to the gold electrode using a hydrophilic linker, and EIS was coupled to the membrane for measuring the conductive properties of proteins. Moreover, other biomarkers such as PSA were detected, using this biomimetic platform, which provided the possibility of studying membrane proteins for cancer diagnosis and producing therapeutic liposomes for treatment.

Similar strategies have also been applied for detecting CTCs. Davies et al. (2012) improved the capture specificity of CTCs by incorporating biomimetic nanoparticles that were camouflaged with a leukocyte membrane and by the microfluidic chip, which reduced the detection time down to 20 min. Anaptamer SYL3C, which could recognize EpCAM^+^CTCs, was functionalized on the magnetic nanoclusters coated with a leukocyte membrane. The complexes were then loaded into microfluidics for CTCs capture.More than 90% of CTCs were captured and detected using this system. Rima et al. (2020) modified immunomagnetic micro/nanoparticles (IMNs) by coating red blood cell (RBC)-derived vesicles, which could dramatically increase the targeting capabilities for CTCs since the vesicle coating eliminated the formation of biomolecule corona covers on the surface of IMNs due to nonspecific adsorption. RBC-IMNs increased the isolation efficiency from 60.22% (no coating) to 95.71% and demonstrated robustness in downstream clinical application by isolating CTCs in 28/30 patients with prostate cancer. Ding et al. (2020) captured CTCs using cancer cell membrane substrate functionalized with bovine serum albumin (BSA) linker and DNA aptamer, targeting the epithelial cell adhesion molecule (EpCAM). Cancer cell membranes can match the surface nanotopography of CTCs perfectly but have low nonspecific binding to other types of cells, leading to highly efficient and specific capture of CTCs. Overall, the specific properties of the membrane could reduce extensive nonspecific binding and improve capture efficiency. This type of technology can also be used for EV detection since EVs contain the same lipid structures, as well as internal and external biomarkers.

Another interesting biomimetic platform originally designed for screening bioactive molecules and their target proteins may also be used for tumor biomarker detection but has not been completed yet. The nano-CaCO_3_ was encapsulated with a cell membrane to form biomimetic nanoparticles, which contained receptors that could bind to bioactive molecules (Ko et al., 2020). The bioactive molecules were then fixed to capture target proteins. The nano-CaCO_3_ biomimetic nanoparticles could serve as a promising tool for protein detection and signaling pathway study once further modifications are carried out.

### Animal/Plant-Inspired Systems

**Figure 4:**
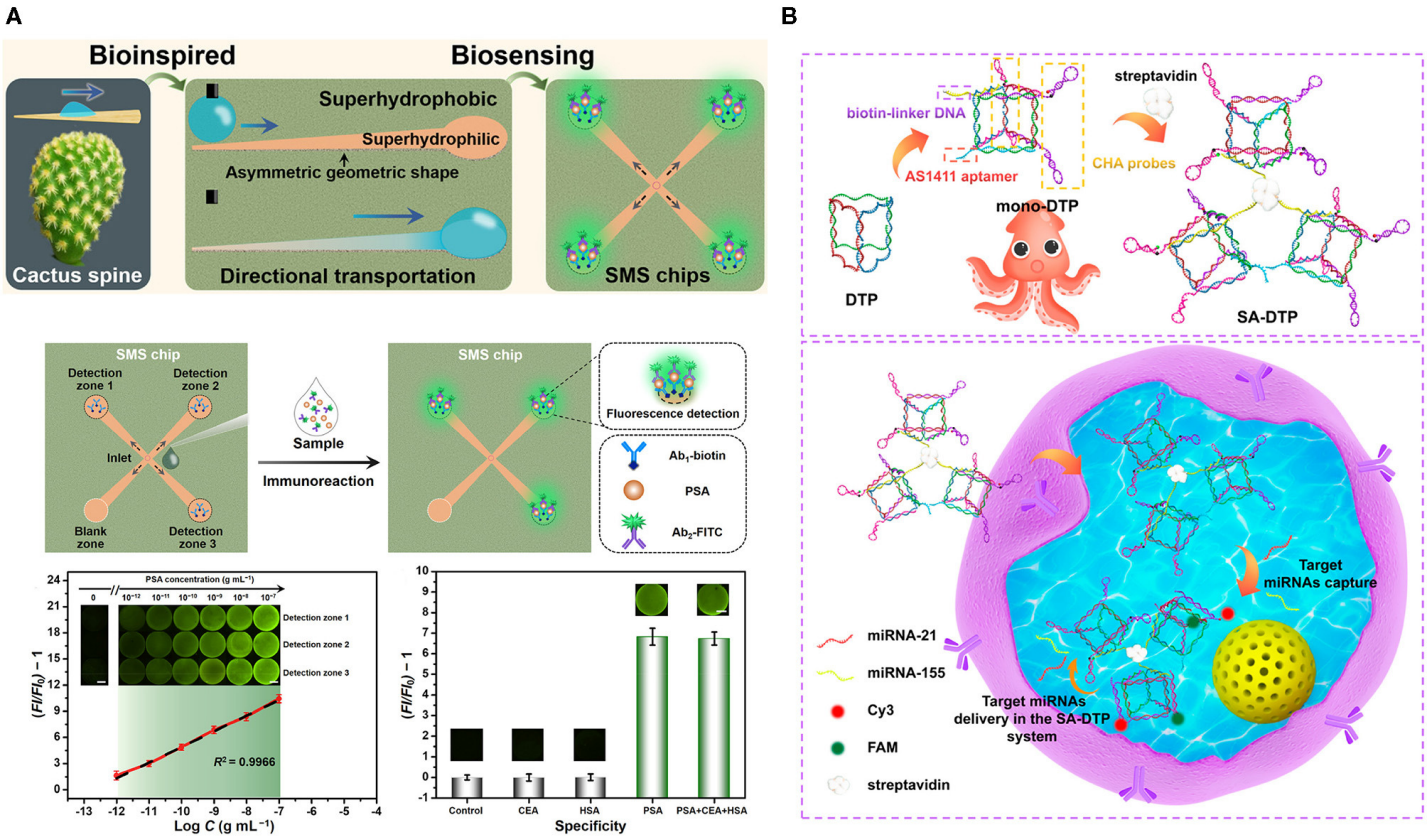
**(A)** Directional droplet transportation on the superwettable microspine (SMS) chip inspired by the cactus spine. Sample [prostate-specific antigen (PSA)] with a detection antibody was loaded at the center and will transport to the detection zone, functionalized with a capture antibody, and the Ab1/PSA/AB2-FITC-generated fluorescence. Sensitivity of the SMS chip with increasing concentration of PSA. Specificity examination of the SMS by setting various conditions, including PSA, PSA+CEA+HAS, HAS, carcinoembryonic antigen (CEA), and negative control (Chen et al., 2020). **(B)** A schematic of fabrication of the SA-DTP, biotin-linker DNA, AS1411 aptamer, and catalytic hairpin assembled (CHA) probes were connected by streptavidin. Below is the multiplex detection of RNA in the living cells (Yang F. et al., 2020). All copyright 2021 American Chemical Society.

Animals and plants have evolved a very extensive variety of different adaptations. Inspired by their special behavior, scientists can potentially improve or modify certain properties of materials, enabling desirable capabilities in biomedical applications (Sun et al., 2018b). For instance, in the open-channel microfluidic system for diagnosis, which requires droplet transportation, many natural behaviors or properties such as Nepenthes alata, spider silk, shorebird beak, and cactus spine have engendered ideas for droplet transportation, leading to different approaches that apply a different gradient of surface energy or the asymmetrical design of the substrate that can drive the droplet (Wu et al., 2019). Chen et al. utilized the behavior of the cactus spine that was capable of directional droplet transportation and fabricated superwettable microspine (SMS) chips with an asymmetrical geometric design, including microchannel and microwell with a superhydrophilic property on the superhydrophobic surface (Gong X. et al., 2020; Guan et al., 2020; Han et al., 2020; Wei D. et al., 2020), which allowed directional droplet transportation by both geometric shape and superhydrophilic property (Figure 4A) (Chen et al., 2020). To better understand the transportation behavior of water droplets, different geometric gradients were measured by the optical contact angle measuring instrument, which revealed that an enlarged gradient facilitates directional droplet transportation. They also studied the effect of geometric asymmetry that could generate Laplace pressure differences on the SMS chip because of the two opposite sides of water droplets, whose larger Laplace pressure differences contributed to the water droplet transportation and reduced the time of movement. Along with the above effects, the superhydrophilicity also improved droplet self-movement. PSA was successfully detected, using the SMS chip. Biofluids contain PSA, and FITC-labeled PSA antibodies were loaded at the inlet of the SMS chip and were transported along microchannels *via* the above-described mechanisms to the detection zone where biotinylated capture antibodies were fixed on the microwells. The detected fluorescence intensity ratio versus the PSA concentration was linearly associated with an over 0.9966 correlation coefficient, and the limit of detection was 1.0–12 g/ml.

Programmable framework nucleic acid (FNA), which is self-assembled DNA scaffolds, can internalize into cells and retain effective functions for a few days, allowing intracellular detection and drug delivery. Yang et al. engineered FNA by composing three mono-DNA triangular prism (DTP), including two pairs of metastable catalytic hairpin assembled (CHA) probes, an AS1411 aptamer, biotin-linker DNA, and mono-DTPs after mixing at room temperature for 3.5 h were combined through streptavidin, which was placed at the center and formed SA-DTP after 0.5-h reaction (Figure 4B) (Yang F. et al., 2020). The whole design was inspired by the tentacles of the octopus, in which the biomimetic SA-DTP served as an octopus to permeate into cells and captured the targeted miRNAs. Then, a specific CHA probe caught the target miRNAs and initiated one group CHA reaction. Then, target miRNAs, after releasing, reacted with adjacent mono-DTPs and initiated more CHA reactions, causing signal amplification for multiplexed detection (miR-155 and miR-21) in MCF-7 breast cancer cells. By applying the specificity of hairpin probes in the form of octopus tentacles, they were able to accelerate the detection process and enhance the signals, leading to high-sensitivity detection of miRNAs.

## Conclusion and Perspectives

Tumor biomarkers present in various human biofluids show great potential in cancer diagnosis and prognosis, but their detection has inevitable biological challenges due to the heterogeneity of cancers and some rare biomarkers. These difficulties have encouraged researchers to develop novel platforms with high capture efficiency, low background noise, effective signal amplification, and short-processing time. Among different technologies, bioinspired or biomimetic materials with unique properties are good candidates for increasing sensitivity and specificity. In this review, we summarize a series of bioinspired platforms for detecting tumor biomarkers. PCs that reflect emission light when light fluorophores overlap with photonic bandgap, combined with immunoaffinity capture or paired DNA aptamer, can dramatically enhance the fluorescence signals. Cell membranes contain numerous nanochannels and lipid structures that are suitable for energy transfer and conversion, and are promising for diagnosis after several modifications, including graphene oxide-fabricated nanochannels, or cell-membrane/vesicles-coated nanoparticles. These cell membrane biomimetic materials can increase capture and targeting efficiency. Bioinspired modified nanomaterials, such as nanoflowers, and nanoenzyme, coupled with different hairpins with hydrogen peroxide, can also enhance the signal. The nanowire mentioned in this review used the idea of intestinal microvilli, which has dramatically increased the surface area for capture. Furthermore, animals and plants can also bring ideas when proposing a platform, such as the above-mentioned cactus spine, which can produce superwettable open microfluidic, leading to high capture efficiency. The octopus-inspired idea in this review is capable of multiplexed analysis.

Besides achievements using bioinspired platforms for driving diagnostics into practice, other aspects, such as automation, data analysis, and high-throughput manufacturing, should be integrated. Until now, all current clinical cancer biomarkers have suffered from relatively low specificity and sensitivity. Hands-on manipulating generally results in low producibility and low repeatability because of unexpected errors, while clinical analysis requires high accuracy. Thus, a programming processing system is necessary. Additionally, developing unsupervised quantification data analysis is essential and time-saving. Machine learning algorithms are suitable for large amounts of data analysis. In addition to sample quantification, machine learning can also be trained for multiplexed biomarker analysis (~ dozens of various cancer-related biomarkers), thus generating fine evaluation metrics when detecting clinical samples of patients, which could increase sensitivity, specificity, and accuracy in practical settings compared with single, dual, or triple biomarker detection. Also, high throughput is another crucial factor; analyzing samples in scale batches simultaneously is essential in a hospital, while the majority of platforms we reviewed here are still proof-of-concept studies, and more cross-validation is necessary.

Another critical factor is to reveal the heterogeneity when using EVs for detection. The above-reviewed methods or other traditional methods (e.g., WB and PCR) are all bulk-based analyses. The structure of EVs may be damaged during the characterization, leading to incomplete information. Thus, developing platforms that guarantee integrity and single-level analysis is a promising research direction.

## Author Contributions

All authors listed have made a substantial, direct and intellectual contribution to the work, and approved it for publication.

## Conflict of Interest

The authors declare that the research was conducted in the absence of any commercial or financial relationships that could be construed as a potential conflict of interest.

## References

[B1] Abubakar M. Figueroa J. Ali H. R. Blows F. Lissowska J. Caldas C.. (2019). Combined quantitative measures of ER., PR, HER2, and KI67 provide more prognostic information than categorical combinations in luminal breast cancer. Mod. Pathol. 32, 1244–1256. 10.1038/s41379-019-0270-4 30976105 PMC6731159

[B2] Akers J. C. Gonda D. Kim R. Carter B. S. Chen C. C. (2013). Biogenesis of extracellular vesicles (EV): exosomes, microvesicles, retrovirus-like vesicles, and apoptotic bodies. J. Neurooncol. 113, 1–11. 10.1007/s11060-013-1084-8 23456661 PMC5533094

[B3] Alix-Panabières C. Pantel K. (2017). Clinical prospects of liquid biopsies. Nat. Biomed. Eng. 1:0065. 10.1038/s41551-017-0065

[B4] Andreu Z. Rivas E. Sanguino-Pascual A. Lamana A. Marazuela M. González-Alvaro I.. (2016). Comparative analysis of EV isolation procedures for miRNAs detection in serum samples. J. Extracell. Vesic. 5:31655. 10.3402/jev.v5.31655 27330048 PMC4916259

[B5] Balk S. P. Ko Y.-J. Bubley G. J. J. J. (2003). Biology of prostate-specific antigen. J. Clin. Oncol. 21, 383–391. 10.1200/JCO.2003.02.083 12525533

[B6] Bast R. C. Jr. Ravdin P. Hayes D. F. Bates S. Fritsche H. Jr. Jessup J. M.. (2001). 2000 update of recommendations for the use of tumor markers in breast and colorectal cancer: clinical practice guidelines of the American Society of Clinical Oncology. J. Clin. Oncol. 19, 1865–1878. 10.1200/JCO.2001.19.6.1865 11251019

[B7] Bi H. Ren D. Zhang J. Wang H. J. Z. (2020). Advances in exosomes in the pathogenesis and diagnosis of lung cancer. Zhongguo Fei Ai Za Zhi 23, 589–596. 10.3779/j.issn.1009-3419.2020.104.18 32702793 PMC7406446

[B8] Bobrie A. Colombo M. Krumeich S. Raposo G. Théry C. (2012). Diverse subpopulations of vesicles secreted by different intracellular mechanisms are present in exosome preparations obtained by differential ultracentrifugation. J. Extracell. Vesic. 1:18397. 10.3402/jev.v1i0.18397 24009879 PMC3760636

[B9] Brock G. Castellanos-Rizaldos E. Hu L. Coticchia C. Skog J. J. T. C.R. (2015). Liquid biopsy for cancer screening, patient stratification and monitoring. Transl. Cancer Res. 4, 280–290. 10.3978/j.issn.2218-676X.2015.06.05

[B10] Chen Y. Li K. Zhang S. Qin L. Deng S. Ge L.. (2020). Bioinspired superwettable microspine chips with directional droplet transportation for biosensing. ACS Nano 14, 4654–4661. 10.1021/acsnano.0c00324 32251583

[B11] Chen Z. Wang Z. Gu Z. J. A. (2019). Bioinspired and biomimetic nanomedicines. Acc. Chem. Res. 52, 1255–1264. 10.1021/acs.accounts.9b00079 30977635 PMC7293770

[B12] Davies R. T. Kim J. Jang S. C. Choi E.-J. Gho Y. S. Park J. (2012). Microfluidic filtration system to isolate extracellular vesicles from blood. Lab Chip. 12, 5202–5210. 10.1039/c2lc41006k 23111789

[B13] Desmond B. J. Dennett E. R. Danielson K. M. J. C. (2020). Circulating extracellular vesicle microrna as diagnostic biomarkers in early colorectal cancer—a review. Cancers 12:52. 10.3390/cancers12010052 31878015 PMC7016718

[B14] Ding P. Wang Z. Wu Z. Zhou Y. Sun N. Pei R. (2020). Natural biointerface based on cancer cell membranes for specific capture and release of circulating tumor cells. ACS Appl. Mater. Interfaces. 12, 20263–20270. 10.1021/acsami.0c03355 32259427

[B15] Dong J. Zhang R. Y. Sun N. Smalley M. Wu Z. Zhou A.. (2019). Bio-inspired nanovilli chips for enhanced capture of tumor-derived extracellular vesicles: toward non-invasive detection of gene alterations in non-small cell lung cancer. ACS Appl. Mater. Interfaces 11, 13973–13983. 10.1021/acsami.9b01406 30892008 PMC6545291

[B16] Dong S. Wang Y. Liu Z. Zhang W. Yi K. Zhang X.. (2020). Beehive-inspired macroporous SERS probe for cancer detection through capturing and analyzing exosomes in plasma. ACS Appl. Mater. Interfaces 12, 5136–5146. 10.1021/acsami.9b21333 31894690

[B17] Dragomir M. Chen B. Calin G. A. (2018). Exosomal lncRNAs as new players in cell-to-cell communication. Transl. Cancer Res. 7(Suppl.2), S243–S52. 10.21037/tcr.2017.10.46 30148073 PMC6107076

[B18] Durrani-Kolarik S. Pool C. A. Gray A. Heyob K. M. Cismowski M. J. Pryhuber G.. (2017). miR-29b supplementation decreases expression of matrix proteins and improves alveolarization in mice exposed to maternal inflammation and neonatal hyperoxia. Am. J. Physiol. Lung Cell. Mol. Physiol. 313, L339–L49. 10.1152/ajplung.00273.2016 28473324 PMC5582933

[B19] Eichelser C. Stückrath I. Müller V. Milde-Langosch K. Wikman H. Pantel K.. (2014). Increased serum levels of circulating exosomal microRNA-373 in receptor-negative breast cancer patients. Oncotarget 5:9650. 10.18632/oncotarget.2520 25333260 PMC4259427

[B20] Foj L. Ferrer F. Serra M. Arévalo A. Gavagnach M. Giménez N.. (2017). Exosomal and non-exosomal urinary miRNAs in prostate cancer detection and prognosis. Prostate 77, 573–583. 10.1002/pros.23295 27990656

[B21] Gong P. Wang Y. Zhang P. Yang Z. Deng W. Sun Z.. (2020). Immunocyte membrane-coated nanoparticles for cancer immunotherapy. Cancers 13:10077. 10.3390/cancers13010077 33396603 PMC7794746

[B22] Gong X. Zhang L. He S. Jiang S. Wang W. Wu Y. (2020). Rewritable superhydrophobic coatings fabricated using water-soluble polyvinyl alcohol. Mater. Des. 196:109112. 10.1016/j.matdes.2020.109112

[B23] Guan F. Song Z. Xin F. Wang H. Yu D. Li G.. (2020). Preparation of hydrophobic transparent paper *via* using polydimethylsiloxane as transparent agent. J. Bioresour. Bioprod. 5, 37–43. 10.1016/j.jobab.2020.03.004

[B24] Han X. Peng J. Jiang S. Xiong J. Song Y. Gong X. (2020). Robust superamphiphobic coatings based on raspberry-like hollow SnO(2) composites. Langmuir 36, 11044–11053. 10.1021/acs.langmuir.0c01923 32856920

[B25] Heimberger A. B. Suki D. Yang D. Shi W. Aldape K. J. J. (2005). The natural history of EGFR and EGFRvIII in glioblastoma patients. J. Transl Med. 3, 1–6. 10.1186/1479-5876-3-38 16236164 PMC1298339

[B26] Heller W. Forkmann G. (1988). Biosynthesis. The Flavonoids. New York, NY: Springer, 399–425. 10.1007/978-1-4899-2913-6_11

[B27] Hessvik N. P. Llorente A. (2018). Current knowledge on exosome biogenesis and release. Cell. Mol. Life Sci. 75, 193–208 10.1007/s00018-017-2595-9 28733901 PMC5756260

[B28] Holt J. K. Noy A. Huser T. Eaglesham D. Bakajin O. J. N. (2004). Fabrication of a carbon nanotube-embedded silicon nitride membrane for studies of nanometer-scale mass transport. ACS Nano 4, 2245–2250. 10.1021/nl048876h

[B29] Hou X. Jiang L. (2009). Learning from nature: building bio-inspired smart nanochannels. ACS Nano 3, 3339–3342. 10.1021/nn901402b 19928930

[B30] Huang C.-S. George S. Lu M. Chaudhery V. Tan R. Zangar R. C.. (2011). Application of photonic crystal enhanced fluorescence to cancer biomarker microarrays. Anal. Chem. 83, 1425–1430. 10.1021/ac102989n 21250635 PMC3039034

[B31] Inoue K. Ohtaka K. (2004). Photonic Crystals: Physics, Fabrication and Applications. New York, NY: Springer Science & Business Media. 10.1007/978-3-540-40032-5

[B32] Iravani S. Varma R. S. J. A. S. C. (2019). Engineering. Plant-derived edible nanoparticles and miRNAs: emerging frontier for therapeutics and targeted drug-delivery. ACS Sustain. Chem. Eng. 7, 8055–8069. 10.1021/acssuschemeng.9b00954

[B33] Işin M. Uysaler E. Özgür E. Köseoglu H. Sanli Ö. Yücel Ö. B.. (2015). Exosomal lncRNA-p21 levels may help to distinguish prostate cancer from benign disease. Front. Genet. 6:168. 10.3389/fgene.2015.00168 25999983 PMC4422020

[B34] Jensen J. L. Maclean G. D. Suresh M. R. Almeida A. Jette D. Lloyd S.. (1991). Possible utility of serum determinations of CA 125 and CA 27.29 in breast cancer management. Int. J. Biol. Markers. 6, 1–6. 10.1177/172460089100600101 1856511

[B35] Kalluri R. (2016). The biology and function of exosomes in cancer. J. Clin. Invest. 126, 1208–1215. 10.1172/JCI81135 27035812 PMC4811149

[B36] Khalil-Mgharbel A. Polena H. Dembélé P. K Hasan Sohag M. M. Alcaraz J.-P. Martin D. K.. (2018). A biomimetic lipid membrane device reveals the interaction of cancer biomarkers with human serum lipidic moieties. Biotechnol. J. 13:1800463. 10.1002/biot.201800463 30457706

[B37] Kim W. J. Bae S. C. J. C. (2008). Molecular biomarkers in urothelial bladder. Cancer 99, 646–652. 10.1111/j.1349-7006.2008.00735.x PMC11160052 18377416

[B38] Ko J. Wang Y. Carlson J. C. T. Marquard A. Gungabeesoon J. Charest A.. (2020). Single extracellular vesicle protein analysis using immuno-droplet digital polymerase chain reaction amplification. Adv. Biosyst. 2020:1900307. 10.1002/adbi.201900307 33274611 PMC8491538

[B39] Kochkodan V. Hilal N. J. D. (2015). A comprehensive review on surface modified polymer membranes for biofouling mitigation. Desalination 356, 187–207. 10.1016/j.desal.2014.09.015

[B40] Koh Y. Q. Almughlliq F. B. Vaswani K. Peiris H. N. Mitchell M. D. (2018). Exosome enrichment by ultracentrifugation and size exclusion chromatography. Front. Biosci. 23, 865–874. 10.2741/4621 28930577

[B41] La Flamme K. E. Popat K. C. Leoni L. Markiewicz E. La Tempa T. J. Roman B. B.. (2007). Biocompatibility of nanoporous alumina membranes for immunoisolation. Biomaterials 28, 2638–2645. 10.1016/j.biomaterials.2007.02.010 17335895 PMC3225223

[B42] Lau A. Halliday C. Chen S. C.-A. Playford E. G. Stanley K. Sorrell T. C. J. J. (2010). Comparison of whole blood, serum, and plasma for early detection of candidemia by multiplex-tandem PCR. J. Clin. Microbiol. 48, 811–816. 10.1128/JCM.01650-09 20042634 PMC2832453

[B43] Lee L. J. Yang Z. Rahman M. Ma J. Kwak K. J. McElroy J.. (2016). Extracellular mRNA detected by tethered lipoplex nanoparticle biochip for lung adenocarcinoma detection. Am. J. Respir. Crit. Care Med. 193, 1431–1433. 10.1164/rccm.201511-2129LE 27304243 PMC4910892

[B44] Li F. Huang J. Ji D. Meng Q. Wang C. Chen S.. (2017). Utility of urinary circulating tumor DNA for EGFR mutation detection in different stages of non-small cell lung cancer patients. Clin. Transl. Oncol. 19, 1283–1291. 10.1007/s12094-017-1669-3 28497422

[B45] Li H.-Y. Liang J.-L. Kuo Y.-L. Lee H.-H. Calkins M. J. Chang H.-T.. (2017). miR-105/93-3p promotes chemoresistance and circulating miR-105/93-3p acts as a diagnostic biomarker for triple negative breast cancer. Breast Cancer Res. 19, 1–14. 10.1186/s13058-017-0918-2 29258605 PMC5738224

[B46] Li M. Zhang Y. Liu Z. Bharadwaj U. Wang H. Wang X.. (2007). Aberrant expression of zinc transporter ZIP4 (SLC39A4) significantly contributes to human pancreatic cancer pathogenesis and progression. Proc. Natl. Acad. Sci. U. S. A. 104, 18636–18641. 10.1073/pnas.0709307104 18003899 PMC2141829

[B47] Li T.-D. Zhang R. Chen H. Huang Z.-P. Ye X. Wang H.. (2018). An ultrasensitive polydopamine bi-functionalized SERS immunoassay for exosome-based diagnosis and classification of pancreatic cancer. Chem. Sci. 9, 5372–5382. 10.1039/C8SC01611A 30009009 PMC6009498

[B48] Liao T. Li X. Tong Q. Zou K. Zhang H. Tang L.. (2017). Ultrasensitive detection of MicroRNAs with morpholino-functionalized nanochannel biosensor. Anal. Chem. 89, 5511–5518. 10.1021/acs.analchem.7b00487 28429595

[B49] Liu H. Jian R. Chen H. Tian X. Sun C. Zhu J.. (2019). Application of biodegradable and biocompatible nanocomposites in electronics: current status and future directions. Nanomaterials 9:70950. 10.3390/nano9070950 31261962 PMC6669760

[B50] Liu Y. Ma Y. Zhang J. Yuan Y. Wang J. (2019). Exosomes: a novel therapeutic agent for cartilage and bone tissue regeneration. Dose-Response 17:1559325819892702. 10.1177/1559325819892702 31857803 PMC6913055

[B51] Lopez C. J. A. M. (2003). Materials aspects of photonic crystals. Adv. Mater. 15, 1679–1704. 10.1002/adma.200300386

[B52] Lv D. Jiao H. Dong J. Sheng L. Liu J. Dong H.. (2019). Biomimetic octopus-like particles for Ultraspecific capture and detection of pathogens. ACS Appl. Mater. Interfaces 11, 22164–22170. 10.1021/acsami.9b05666 31149791

[B53] Ma C. Jiang F. Ma Y. Wang J. Li H. Zhang J. (2019). Isolation and detection technologies of extracellular vesicles and application on cancer diagnostic. Dose-Response 17:1559325819891004. 10.1177/1559325819891004 31839757 PMC6902397

[B54] Ma Y. Dong S. Li X. Kim B. Y. S. Yang Z. Jiang W. (2021). Extracellular vesicles: an emerging nanoplatform for cancer therapy. Front. Oncol. 10:606906. 10.3389/fonc.2020.606906 33628730 PMC7897670

[B55] Madhavan B. Yue S. Galli U. Rana S. Gross W. Müller M.. (2015). Combined evaluation of a panel of protein and miRNA serum-exosome biomarkers for pancreatic cancer diagnosis increases sensitivity and specificity. Int. J. Cancer. 136, 2616–2627. 10.1002/ijc.29324 25388097

[B56] Maheswaran S. Sequist L. V. Nagrath S. Ulkus L. Brannigan B. Collura C. V.. (2008). Detection of mutations in EGFR in circulating lung-cancer cells. N. Engl. J. Med. 359, 366–377. 10.1056/NEJMoa0800668 18596266 PMC3551471

[B57] Mann D. Edwards R. Ho S. Lau W. Glazer G. J. E. (2000). Elevated tumour marker CA19-9: clinical interpretation and influence of obstructive jaundice. Eur. J. Surg. Oncol. 26, 474–479. 10.1053/ejso.1999.0925 11016469

[B58] Minciacchi V. R. Freeman M. R. Di Vizio D. (2015). Extracellular vesicles in cancer: exosomes, microvesicles and the emerging role of large oncosomes. Semin Cell Dev. Biol. 40, 41–51. 10.1016/j.semcdb.2015.02.010 25721812 PMC4747631

[B59] Mohanpuria P. Rana N. K. Yadav S. K. J. J. (2008). Biosynthesis of nanoparticles: technological concepts and future applications. J. Nanoparticle Res. 10, 507–517. 10.1007/s11051-007-9275-x

[B60] Monteiro J. P. de Oliveira J. H. Radovanovic E. Brolo A. G. Girotto E. M. (2016). Microfluidic plasmonic biosensor for breast cancer antigen detection. Plasmonics 11, 45–51. 10.1007/s11468-015-0016-127997366

[B61] Moss E. Hollingworth J. Reynolds T. J. J. (2005). The role of CA125 in clinical practice. J. Clin. Pathol. 58, 308–312. 10.1136/jcp.2004.018077 15735166 PMC1770590

[B62] Muller L. Muller-Haegele S. Mitsuhashi M. Gooding W. Okada H. Whiteside T. L. J. O. (2015). Exosomes isolated from plasma of glioma patients enrolled in a vaccination trial reflect antitumor immune activity and might predict survival. Oncoimmunology 4:e1008347. 10.1080/2162402X.2015.1008347 26155415 PMC4485717

[B63] Naik R. R. Singamaneni S. J. C. (2017). Introduction: bioinspired and biomimetic materials. Chem. Rev. 117, 12581–12583. 10.1021/acs.chemrev.7b00552 29065691

[B64] Parker A. R. Townley HEJNn. (2007). Biomimetics of photonic nanostructures. Nat. Nanotech 2, 347–353. 10.1038/nnano.2007.152 18654305

[B65] Patel G. K. Khan M. A. Zubair H. Srivastava S. K. Khushman Md. Singh S.. (2019). Comparative analysis of exosome isolation methods using culture supernatant for optimum yield, purity and downstream applications. Sci. Rep. 9:5335. 10.1038/s41598-019-41800-2 30926864 PMC6441044

[B66] Pavlou M. P. Diamandis E. Blasutig I. J. C. (2013). The long journey of cancer biomarkers from the bench to the clinic. Clin. Chem. 59, 147–157. 10.1373/clinchem.2012.184614 23019307

[B67] Peng X. Wen Z.-B. Yang P. Chai Y.-Q. Liang W.-B. Yuan R. (2019). Biomimetic 3D DNA nanomachine *via* free DNA walker movement on lipid bilayers supported by hard SiO_2_@CdTe nanoparticles for ultrasensitive MicroRNA detection. Analyt. Chem. 91, 14920–14926. 10.1021/acs.analchem.9b03263 31674756

[B68] Pratsinis A. Kelesidis G. A. Zuercher S. Krumeich F. Bolisetty S. Mezzenga R.. (2017). Enzyme-mimetic antioxidant luminescent nanoparticles for highly sensitive hydrogen peroxide biosensing. ACS Nano 11, 12210–12218. 10.1021/acsnano.7b05518 29182310

[B69] Que R. Ding G. Chen J. Cao L. (2013). Analysis of serum exosomal microRNAs and clinicopathologic features of patients with pancreatic adenocarcinoma. World J. Surg. Oncol. 11:219. 10.1186/1477-7819-11-219 24007214 PMC3766671

[B70] Reátegui E. Aceto N. Lim E. J. Sullivan J. P. Jensen A. E. Zeinali M.. (2015). Tunable nanostructured coating for the capture and selective release of viable circulating tumor cells. Adv. Mater. 27, 1593–1599. 10.1002/adma.201404677 25640006 PMC4492283

[B71] Riethdorf S. Fritsche H. Müller V. Rau T. Schindlbeck C. Rack B.. (2007). Detection of circulating tumor cells in peripheral blood of patients with metastatic breast cancer: a validation study of the CellSearch system. Clin. Cancer Res. 13, 920–928. 10.1158/1078-0432.CCR-06-1695 17289886

[B72] Rima X. Y. Walters N. Nguyen L. T. H. Reátegui E. (2020). Surface engineering within a microchannel for hydrodynamic and self-assembled cell patterning. Biomicrofluidics 14:014104. 10.1063/1.5126608 31933714 PMC6941948

[B73] Rissin D. M. Kan C. W. Campbell T. G. Howes S. C. Fournier D. R. Song L.. (2010). Single-molecule enzyme-linked immunosorbent assay detects serum proteins at subfemtomolar concentrations. Nat. Biotechnol. 28, 595–599. 10.1038/nbt.1641 20495550 PMC2919230

[B74] Rönnstrand L. Lennartsson J. J. A. (2016). Oncology Ci Haematology. KIT (v-kit Hardy-Zuckerman 4 feline sarcoma viral oncogene homolog). Philadelphia, PA: American Association for Cancer Research (AACR).

[B75] Sandfeld-Paulsen B. Jakobsen K. R. Bæk R. Folkersen B. H. Rasmussen T. R. Meldgaard P.. (2016). Exosomal proteins as diagnostic biomarkers in lung cancer. J. Thoracic Oncol. 11, 1701–1710. 10.1016/j.jtho.2016.05.034 27343445

[B76] Shi C. Xie H. Ma Y. Yang Z. Zhang J. (2020). Nanoscale technologies in highly sensitive diagnosis of cardiovascular diseases. Front. Bioeng. Biotechnol. 8:531. 10.3389/fbioe.2020.00531 32582663 PMC7289988

[B77] Shi J. F. Ma Y. F. Zhu J. Chen Y. X. Sun Y. T. Yao Y. C.. (2018). A review on electroporation-based intracellular delivery. Molecules 23:113044. 10.3390/molecules23113044 30469344 PMC6278265

[B78] Sokolova V. Ludwig A.-K. Hornung S. Rotan O. Horn P. A. Epple M.. (2011). Characterisation of exosomes derived from human cells by nanoparticle tracking analysis and scanning electron microscopy. Coll. Surf. B Biointerfaces 87, 146–150. 10.1016/j.colsurfb.2011.05.013 21640565

[B79] Spano J.-P. Lagorce C. Atlan D. Milano G. Domont J. Benamouzig R.. (2005). Impact of EGFR expression on colorectal cancer patient prognosis and survival. Ann. Oncol. 16, 102–108. 10.1093/annonc/mdi006 15598946

[B80] Sun J. Wang X. Wu J. Jiang C. Shen J. Cooper M. A.. (2018b). Biomimetic moth-eye nanofabrication: enhanced antireflection with superior self-cleaning characteristic. Sci. Rep. 8:5438. 10.1038/s41598-018-23771-y 29615712 PMC5883013

[B81] Sun J. Zhao Y. Yang Z. Shen J. Cabrera E. Lertola M. J.. (2018a). Highly stretchable and ultrathin nanopaper composites for epidermal strain sensors. Nanotechnology 29:355304. 10.1088/1361-6528/aacc59 29897348

[B82] Sun X. Yu K. Zhou Y. Dong S. Hu W. Sun Y.. (2021). Self-assembled pH-sensitive polymeric nanoparticles for the inflammation-targeted delivery of Cu/Zn-superoxide dismutase. ACS Appl. Mater. Interfaces 13, 18152–18164. 10.1021/acsami.1c03589 33764751

[B83] Tan X. Broses L. J. Zhou M. Day K. C. Liu W. Li Z.. (2020). Multiparameter urine analysis for quantitative bladder cancer surveillance of orthotopic xenografted mice. Lab Chip 20, 634–646. 10.1039/C9LC01006H 31922156

[B84] Tan X. Chen Q. Zhu H. Gong Y. Chen Y.-C. Li X.. (2019). A fast and reproducible ELISA laser platform. Int. Soc. Opt. Photon. 12:2057517. 10.1117/12.250751731829015

[B85] Tan X. David A. Day J. Tang H. Dixon E. R. Zhu H.. (2018). Rapid mouse follicle stimulating hormone quantification and estrus cycle analysis using an automated microfluidic chemiluminescent ELISA system. ACS Sensors 3, 2327–2334. 10.1021/acssensors.8b00641 30335974 PMC6533910

[B86] Tan X. Day K. C. Li X. Broses L. J. Xue W. Wu W.. (2021). Quantification and immunoprofiling of bladder cancer cell-derived extracellular vesicles with microfluidic chemiluminescent ELISA. Biosens. Bioelectr. 2021:100066. 10.1016/j.biosx.2021.100066

[B87] Toraih E. A. Abdallah H. Y. Rashed E. A. El-Wazir A. Tantawy M. A. Fawzy M. S. J. E. (2019). Comprehensive data analysis for development of custom qRT-PCR miRNA assay for glioblastoma: a prevalidation study. Epigenomics 11, 367–380. 10.2217/epi-2018-0134 30793921

[B88] Tran T. D. Kim M. I. (2018). Organic-inorganic hybrid nanoflowers as potent materials for biosensing and biocatalytic applications. BioChip J. 12, 268–279. 10.1007/s13206-018-2409-7

[B89] Vaidyanathan R. Soon R. H. Zhang P. Jiang K. Lim C. T. J. L. C. (2019). Cancer diagnosis: from tumor to liquid biopsy and beyond. Lap Chip 19, 11–34. 10.1039/C8LC00684A 30480287

[B90] Veziroglu E. M. Mias G. I. (2020). Characterizing extracellular vesicles and their diverse RNA contents. Front. Genet. 11:700. 10.3389/fgene.2020.00700 32765582 PMC7379748

[B91] Walters N. Nguyen L. T. H. Zhang J. Shankaran A. Reátegui E. (2019). Extracellular vesicles as mediators of *in vitro* neutrophil swarming on a large-scale microparticle array. Lab Chip 19, 2874–2884. 10.1039/C9LC00483A 31343025

[B92] Walters N. Rima X. Y. Zhang J. Nguyen L. T. Germain R. N. Lämmermann T.. (2021). Analyzing inter-leukocyte communication and migration *in vitro*: neutrophils play an essential role in monocyte activation during swarming. Front. Immunol. 12:1550. 10.3389/fimmu.2021.671546 34054848 PMC8152805

[B93] Wang Y. Li Z. Lin Q. Wei Y. Wang J. Li Y.. (2019). Highly sensitive detection of bladder cancer-related miRNA in urine using time-gated luminescent biochip. ACS Sensors 4, 2124–2130. 10.1021/acssensors.9b00927 31313911

[B94] Wang Y.-H. Ji J. Wang B.-C. Chen H. Yang Z.-H. Wang K.. (2018). Tumor-derived exosomal long noncoding RNAs as promising diagnostic biomarkers for prostate cancer. Cell. Physiol. Biochem. 46, 532–545. 10.1159/000488620 29614511

[B95] Wei D. W. Wei H. Gauthier A. C. Song J. Jin Y. Xiao H. (2020). Superhydrophobic modification of cellulose and cotton textiles: methodologies and applications. J. Bioresourc. Bioprod. 5, 1–15. 10.1016/j.jobab.2020.03.001

[B96] Wei X. Bian F. Cai X. Wang Y. Cai L. Yang J.. (2020). Multiplexed detection strategy for bladder cancer MicroRNAs based on photonic crystal barcodes. Analyt. Chem. 92, 6121–6127. 10.1021/acs.analchem.0c00630 32227890

[B97] Willms E. Cabañas C. Mäger I. Wood M. J. A. Vader P. (2018). Extracellular vesicle heterogeneity: subpopulations, isolation techniques, and diverse functions in cancer progression. Front. Immunol. 9:738. 10.3389/fimmu.2018.00738 29760691 PMC5936763

[B98] Wu C. Du Y.-W. Huang L. Ben-Shoshan Galeczki Y. Dagan-Wiener A. Naim M.. (2017). Biomimetic sensors for the senses: towards better understanding of taste and odor sensation. Sensors 17:2881. 10.3390/s17122881 29232897 PMC5750803

[B99] Wu T. Yang Y. Cao Y. Song Y. Xu L.-P. Zhang X.. (2018). Bioinspired DNA–inorganic hybrid nanoflowers combined with a personal glucose meter for onsite detection of miRNA. ACS Appl. Mater. Interfaces 10, 42050–42057. 10.1021/acsami.8b15917 30457317

[B100] Wu Y. Feng J. Gao H. Feng X. Jiang L. J. A. M. (2019). Superwettability-based interfacial chemical reactions. Adv. Mater. 31:1800718. 10.1002/adma.201800718 30592333

[B101] Wu Z. Ma X. Ma Y. Yang Z. Yuan Y. Liu C. (2020). Core/Shell PEGS/HA hybrid nanoparticle *via* micelle-coordinated mineralization for tumor-specific therapy. ACS Appl. Mater. Interfaces 12, 12109–12119. 10.1021/acsami.0c00068 32068397

[B102] Yamashita T. Takahashi Y. Nishikawa M. Takakura Y. (2016). Effect of exosome isolation methods on physicochemical properties of exosomes and clearance of exosomes from the blood circulation. Eur. J. Pharmaceut. Biopharmaceut. 98, 1–8. 10.1016/j.ejpb.2015.10.017 26545617

[B103] Yáñez M. ó. M. Siljander P. R. M. Andreu Z. Bedina Zavec A. Borràs F. E. Buzas E. I.. (2015). Biological properties of extracellular vesicles and their physiological functions. J. Extracell. Vesicl. 4:27066. 10.3402/jev.v4.27066 25979354 PMC4433489

[B104] Yang F. Cheng Y. Zhang Y. Wei W. Dong H. Lu H.. (2020). Bioinspired framework nucleic acid capture sensitively and rapidly resolving MicroRNAs biomarkers in living cells. Analyt. Chem. 92, 4411–4418. 10.1021/acs.analchem.9b05304 32056432

[B105] Yang X. Ma Y. Xie H. Dong S. Rao G. Yang Z.. (2021). Extracellular vesicles in the treatment of Parkinson's disease: a review. Curr. Med. Chem. 2021:2174. 10.2174/0929867328666210113170941 33441061

[B106] Yang Z. Ma Y. Zhao H. Yuan Y. Kim B. Y. S. (2020a). Nanotechnology platforms for cancer immunotherapy. WIREs Nanomed. Nanobiotechnol. 12:e1590. 10.1002/wnan.1590 31696664

[B107] Yang Z. Shi J. Xie J. Wang Y. Sun J. Liu T.. (2020b). Large-scale generation of functional mRNA-encapsulating exosomes *via* cellular nanoporation. Nat. Biomed. Eng. 4, 69–83. 10.1038/s41551-019-0485-1 31844155 PMC7080209

[B108] Yang Z. Xie J. Zhu J. Kang C. Chiang C. Wang X.. (2016). Functional exosome-mimic for delivery of siRNA to cancer: *in vitro* and *in vivo* evaluation. J. Control Rel. 243, 160–171. 10.1016/j.jconrel.2016.10.008 27742443

[B109] Yoo C. E. Kim G. Kim M. Park D. Kang H. J. Lee M.. (2012). A direct extraction method for microRNAs from exosomes captured by immunoaffinity beads. Analyt. Biochem. 431, 96–98. 10.1016/j.ab.2012.09.008 22982508

[B110] Yuana Y. Levels J. Grootemaat A. Sturk A. Nieuwland R. (2014). Co-isolation of extracellular vesicles and high-density lipoproteins using density gradient ultracentrifugation. J. Extracell. Vesicl. 3:10.3402/jev.v3.23262. 10.3402/jev.v3.23262 25018865 PMC4090368

[B111] Zhang J. Nguyen L. T. H. Hickey R. Walters N. Wang X. Kwak K. J.. (2021). Immunomagnetic sequential ultrafiltration (iSUF) platform for enrichment and purification of extracellular vesicles from biofluids. Sci. Rep. 11:8034. 10.1038/s41598-021-86910-y 33850163 PMC8044115

[B112] Zhang J. Zhu Y. Shi J. Zhang K. Zhang Z. Zhang H. (2020). Sensitive signal amplifying a diagnostic biochip based on a biomimetic periodic nanostructure for detecting cancer exosomes. ACS Appl. Mater. Interfaces 12, 33473–33482. 10.1021/acsami.0c06785 32603586

[B113] Zhang S. Cheng J. Shi W. Li K.-B. Han D.-M. Xu J.-J. (2020). Fabrication of a biomimetic nanochannel logic platform and its applications in the intelligent detection of miRNA related to liver cancer. Analyt. Chem. 92, 5952–5959. 10.1021/acs.analchem.0c00147 32207618

[B114] Zhang W. Xia W. Lv Z. Ni C. Xin Y. Yang L. (2017). Liquid biopsy for cancer: circulating tumor cells, circulating free DNA or exosomes? Cell Physiol. Biochem. 41, 755–768. 10.1159/000458736 28214887

[B115] Zhang X. Yuan X. Shi H. Wu L. Qian H. Xu W. (2015). Exosomes in cancer: small particle, big player. J. Hematol. Oncol. 8:83. 10.1186/s13045-015-0181-x 26156517 PMC4496882

[B116] Zhang X. Zhang F. Zhu F. Zhang X. Tian D. Johnson R. P.. (2019). Bioinspired γ-cyclodextrin pseudorotaxane assembly nanochannel for selective amino acid transport. ACS Appl. Bio Mater. 2, 3607–3612. 10.1021/acsabm.9b00473 35030747

[B117] Zhao X.-P. Liu F.-F. Hu W.-C. Younis M. R. Wang C. Xia X.-H. (2019). Biomimetic nanochannel-ionchannel hybrid for ultrasensitive and label-free detection of MicroRNA in cells. Analyt. Chem. 91, 3582–3589. 10.1021/acs.analchem.8b05536 30758184

[B118] Zhou H. Yuen P. S. Pisitkun T. Gonzales P. A. Yasuda H. Dear J. W.. (2006). Collection, storage, preservation, and normalization of human urinary exosomes for biomarker discovery. Kidney Int. 69, 1471–1476. 10.1038/sj.ki.5000273 16501490 PMC2276656

[B119] Zhu J.-Y. Zheng D.-W. Zhang M.-K. Yu W.-Y. Qiu W.-X. Hu J.-J.. (2016). Preferential cancer cell self-recognition and tumor self-targeting by coating nanoparticles with homotypic cancer cell membranes. Nano Lett. 16, 5895–5901. 10.1021/acs.nanolett.6b02786 27513184

